# Factor Analysis Affecting Degree of Depression in Family Caregivers of Patients with Spinal Cord Injury: A Cross-Sectional Pilot Study

**DOI:** 10.3390/ijerph191710878

**Published:** 2022-08-31

**Authors:** Su-Jin Lee, Myung-Gwan Kim, Jung hee Kim, Yu-Sun Min, Chul-Hyun Kim, Kyoung-Tae Kim, Jong-Moon Hwang

**Affiliations:** 1 Graduate School of Public Health, Kyungpook National University, Daegu 41944, Korea; 2 Department of Biomedical Informatics, CHA University School of Medicine, Seongnam 13415, Korea; 3 Institute for Biomedical Informatics, CHA University School of Medicine, Seongnam 13415, Korea; 4 College of Pharmacy, Industrial Pharmacy, Chungbuk National University, Cheongju-si 28644, Korea; 5 Department of Rehabilitation Medicine, School of Medicine, Kyungpook National University, Daegu 41944, Korea; 6 Department of Rehabilitation Medicine, Kyungpook National University Chilgok Hospital, Daegu 41404, Korea; 7 Department of Rehabilitation Medicine, Kyungpook National University Hospital, Daegu 41944, Korea; 8 Department of Neurosurgery, Kyungpook National University Hospital, Daegu 41944, Korea; 9 Department of Neurosurgery, School of Medicine, Kyungpook National University, Daegu 41944, Korea

**Keywords:** spinal cord injuries, caregivers, caregiver-family, family caregiver, caregiver-spouse, caregiver burden, depression

## Abstract

This study was conducted to determine the degree of depression in family caregivers of spinal-cord-injury patients and to identify factors influencing family caregivers’ depression. The final study subjects were 30 (family caregivers: 6 males and 24 females). The CES-D of family caregivers; general characteristics of spinal-cord-injury patients and family caregivers; and information on physical health, household income, leisure, social activity, family relationship, and life-in-general status of family caregiver were collected. A frequency analysis, normality test, Mann–Whitney test, Kruskal–Wallis test, Spearman Correlation analysis, hierarchical regression analysis, and spider network through a path model analysis were performed. As for the general characteristics, when the patient was economically active, the caregiver’s depression was mean ± SD; 2.04 ± 0.71; otherwise, it was mean ± SD 2.86 ± 0.74, indicating that the caregiver of the non-economic activity patient was more depressed (*p* = 0.013). In Model 1 of the multiple regression analysis to understand the effect on the depression of the caregiver, it was confirmed that the depression of the caregiver decreased as the family caregiver had more leisure and social activities (B = −0.718, *p* = 0.001). In Model 2, it was found that the depression of caregivers increased when the patient did not engage in economic activity (B = 0.438, *p* = 0.016). In the spider-web form through the path model analysis, as the family’s economic level increased, physical health increased by B = 0.755 (*p* < 0.001), and the increase in physical health (B = 0.424, *p* = 0.042) was, in turn, a factor in the increase of engagement in leisure and social activities. Various policies will be needed for the successful return to society of spinal-cord-injury patients by ensuring that their leisure and social activities and establishing measures to support their economical income.

## 1. Introduction

Individuals with spinal cord injury (SCI) are classified as severely disabled and experience multiple disabilities due to nerve damage caused by sports accidents, traffic accidents, or trauma injuries at work [1]. Despite the development of modern medicine and treatment technology, patients with SCI are often prevented from further exacerbating their symptoms; however, there are limitations, and the duration of the illness can be prolonged [2]. Since the 1970s, the lifespan of individuals with spinal cord disorders has been steadily increasing, and more than 80% of patients with spinal cord disabilities enjoy lifespans close to those of non-disabled people [3]. In this sense, continuous management of these patients is needed, and as the hospitalization period is shortened and outpatient treatment is increasing, the importance of the family as a guardian and caregiver is emphasized in providing appropriate care for the patients with SCI [4].

When a family directly helps a person with SCI in daily life, family members experience continuous psychological and physical pressure [5,6]. In addition to physical function, individuals with SCI experience repeated long-term difficulties due to sensory disturbances, bowel and urinary disorders, and hospitalization and outpatient treatment. Due to the characteristics of individuals disabled with SCI, special medical services are required, and families face various difficulties that cannot be solved with existing family support policies [6]. As family caregivers are inevitably subjected to constant psychological pressure, they suffer from severe emotional changes, depressive symptoms, sleep disorders, eating disorders, and difficulties in interpersonal relationships [7]. Therefore, spinal cord disorders are considered “family affairs” because the effects of an accident or disease are not limited to the person concerned but also have serious psychosocial effects on the family [8]. In addition to these physical and psychological difficulties, economic activities are impossible or restricted for the patient and the family after the SCI, which aggravates economic difficulties, the burden of housework, concerns about raising children, and changes in marital relationships [9]. This problem can lead to the dissolution of a family [10]. From this perspective, several existing studies have described the relationship between the care burden and depression of various types of family caregivers by using the Center for Epidemiology Studies Depression Scale (CES-D) [11,12,13,14]. 

In a study that used the CES-D to measure the degree of depression in caregivers with intellectual disabilities, the burden of care tended to be higher among female caregivers, unemployed individuals, parents, cohabitants, and caregivers with health problems. Family-caregiver characteristics, such as perceptions of spousal relationships, health problems, and care time and cost, were associated with depression [13]. In a study of 51 caregivers of dementia patients, approximately 64.7% of participants showed depressive symptoms (CES-D score ≥ 16), and these symptoms increased significantly during the 3-month follow-up period [14]. A study on the depression of caregivers of stroke survivors demonstrated that the relationship with the patient, the burden of “care”, and depression had a significant effect on their quality of life [11]. Hence, it is necessary to develop and implement an intervention program that can systematically supplement care-related characteristics and alleviate the burden of support and depression.

Existing studies on families with severely disabled individuals are mainly focused on those caring for individuals with developmental disabilities; most studies have targeted mothers who are the primary caregivers of children with developmental disabilities, family guardians of terminal cancer patients, and family guardians of dementia patients. However, these studies did not include families of individuals with severe physical disabilities [7]. Therefore, it is worthwhile to conduct research on families of individuals with SCI. 

Therefore, the purpose of this study was to understand the influence of demographic and quality-of-life factors on the level of depression in family caregivers supporting individuals with SCI and to establish a family support system for these caregivers.

## 2. Material and Methods

### 2.1. Study Design and Population

The demographic characteristics (e.g., age, sex, educational background, economic activity, current marital status, and religion) of SCI patients and their family caregiver were evaluated. In addition, the general care-related characteristics of family caregivers (e.g., relationship with the patient and whether nursing care has changed), as well as their degree of life satisfaction (e.g., physical health, family economic level, leisure and social activities, family relationships, and life in general) and their degree of depression, were investigated. 

This cross-sectional study analyzed primary data. The description of the study was prepared according to the STROBE reporting guidelines (https://www.strobe-statement.org/) (accessed on 17 January 2022). The degree of depression in family caregivers of patients with SCI was set as a dependent variable. This was to determine what kind of relationship exists among characteristics, physical health, family economic level, leisure and social activities, and family relationships.

A questionnaire survey was conducted targeting 33 family caregivers of patients hospitalized for SCI at the Department of Rehabilitation Medicine at K University Hospital, located in Region D of Korea. For recruitment, the attending physician first explained the study to the patient and guardian, and those who agreed to the study orally informed the researcher of their intention to participate. From 21 October 2021 to 30 November 2021, separate questionnaires were provided to the caregiver and patients. Caregiver and patient surveys were conducted in separate rooms. Two researchers, who received sufficient training to conduct the survey, explained the purpose of the study directly to the survey participants in their own separate spaces, and after obtaining written consent, data were collected by using an anonymous structured questionnaire. All 33 questionnaires were retrieved; however, a total of 30 participants were included for the final analysis, after excluding three questionnaires that could not be analyzed due to incomplete responses. The study was conducted according to the guidelines of the Declaration of Helsinki and approved by the Clinical Trial Committee of Kyungpook National University Hospital (IRB No.2021-08-034).

### 2.2. Dependent Variables

#### Center for Epidemiologic Studies Depression Scale

The CES-D [15], a depression measurement tool developed by Radloff (1977), consists of 20 items that measure depression and emotion. The depressive index of caregivers of patients with SCI was measured by using the research tool developed by Kyum-Koo Chon [16] in the Korean version of the CES-D. Each item in the Korean version of the CES-D is answered with “rarely (1 day or less)” to “mostly (5–7 days)” (as the maximum score) to the question, “Please choose the one that best expresses how you felt and acted in the past week”; the higher the score, the higher the degree of depression. The subfactors included depressive emotions (Factor I), positive emotions (Factor II), interpersonal failure (Factor III), and physical deterioration (Factor IV). Based on the explanatory power of the eigenvalues of each subfactor, the average score for all tools showed an explanatory power of 57.2%. The reliability of the tool measured at the time of tool development was 0.89 for internal consistency (Cronbach’s α). In this study, each of the 20 items of the Korean version of CES-D was composed of a Likert scale, using the number of items (4 times “I felt good”, 8 times “I felt hopeful”, 12 times “I was happy”, and 16 times “I enjoyed life”). Scores were calculated in reverse order. In this study, Cronbach’s coefficient, which indicates internal reliability, was 0.87. 

### 2.3. Independent Variables

#### 2.3.1. Demographic Characteristics 

Age, gender, educational background, economic activity, marital status, and religion were investigated as variables representing the sociodemographic characteristics of SCI patients and their family caregivers. Open-ended responses to age were classified as under 60 years old and over 60 years old; gender as male or female; and educational background as junior-high-school graduation or less, high-school graduation, or university graduation or higher. In this study, “economic activity” was measured by the question, “Are you currently engaged in an economic activity that generated a certain amount of income?” The two answers were defined as “‘Yes” and “No”. Marital status was defined as single, married, or other (such as separated, widowed), and religion as “exists” and “does not exist”.

#### 2.3.2. General Characteristics of Family Caregivers Related to Care

The general characteristics of family caregivers related to care were classified into a spouse, parent, child, or others (grandparents, grandchildren, brother, and sister) in response to the question of “relationship with the SCI patient currently caring for”. The remaining questions were classified with “yes” or “no” responses.

#### 2.3.3. Life Satisfaction of Families with Spinal Cord Disabilities 

The items on life satisfaction of families with spinal cord disabilities consisted of questions about the difficulties and life satisfaction as reported by Seo et al. [7]. Of the six items, the degree of subjective satisfaction felt by participants was used concerning physical health, family economics, social activities (such as leisure), family relationships, and overall life satisfaction, except for mental health [7]. Each question was answered on a 4-point scale: “very satisfied” (4 points), “satisfied” (3 points), “dissatisfied” (2 points), and “very dissatisfied” (1 point). In this study, Cronbach’s coefficient, which indicates internal reliability, was 0.92. 

### 2.4. Statistical Analyses

All statistical analyses were performed by using R studio (R version 4.1.2), an open-source statistical analysis software package. The statistical significance level for the rejection of the null hypothesis was set to less than 0.05.

A frequency analysis was performed to confirm the distribution of the general characteristics of caregivers and patients. The minimum and maximum values, mean, and standard deviation were analyzed through descriptive statistical analysis to confirm the distribution of caregiver depression, physical health, family economic level, leisure and social activities, family relationships, and life satisfaction. Overall, skewness did not exceed 2 as an absolute value, and kurtosis did not exceed 4; therefore, there was no problem with the normality of the data. However, because the sample size in our study was less than 30, a nonparametric test was selected as the overall analysis technique

The Mann–Whitney test and Kruskal–Wallis test (nonparametric test methods) were used to check the distribution of depression, physical health, family economic level, leisure and social activities, family relationships, and overall life satisfaction of caregivers according to the characteristics of caregivers and patients. Spearman’s correlation analysis, another nonparametric test method, was performed to determine the correlation between the guardian’s physical health, family economic level, leisure and social activities, family relationships, overall life satisfaction in general, and depression.

A hierarchical multiple regression analysis was performed to identify the factors affecting caregivers’ depression, and pathway analysis was performed to determine the path influencing the depression.

## 3. Results

### 3.1. General Characteristics (Caregivers and Patients)

In regard to the general characteristics of the caregiver, the age group under 60 years old was the highest, at 63.3%, the gender was female (80.0%), and the education level was the same in all three groups. As for economic activity, 56.7% of the group answered “No”, 83.3% of the “married” group of married status, 60.0% of the religious group, 63.3% of “spouse” about the relationship with the patient, and “spouse” of the patient care shifting (Table 1).

The general characteristics of the patient were 60 years or older (60.0%), male (83.3%), high school graduate (43.3%), economic activity “No” (63.3%), “Single” 76.7%, and “No religion” 56.7% (Table 1).

### 3.2. Depression, Physical Health, Household Income, Leisure and Social Activity, Family Relations, and Overall Life Satisfaction in Caregivers

The average values of the indicators for caregivers were as follows: depression, 2.56 ± 0.82; physical health, 2.70 ± 0.79; family economic level, 2.70 ± 0.75; leisure and social activities, 2.50 ± 0.86; family relationships, 2.90 ± 0.76; and overall life satisfaction, 2.50 ± 0.86. (Table 2).

### 3.3. Comparison of Caregiver’s Depression, Physical Health, Household Income, Leisure and Social Activity, Family Relations, Overall Life Satisfaction and General Characteristics (Caregivers and Patients)

The first variable that showed a difference in caregivers’ depression among all general characteristics was the patient’s economic activity. When patients were economically active, caregivers’ depression was 2.04 ± 0.71 (mean ± SD); otherwise, it was 2.86 ± 0.74 (mean ± SD), which indicated that caregivers of inactive patients who do not have income were more depressed (*p* = 0.013). Other variables that revealed differences in physical health were the caregivers’ gender and economic activity. When caregivers were men, physical health was 3.17 ± 0.81 (mean ± SD), while it was 2.55 ± 0.72 (mean ± SD) for women; thus, physical health was better when caregivers were male (*p* = 0.027). When caregivers were economically active, physical health was 3.08 ± 0.049 (mean ± SD), and when they were not, physical health was 2.41 ± 0.87 (mean ± SD) (*p* = 0.022). Regarding scores for household income, leisure, social activities, and family relationships of caregivers, no variables showed significant differences. Finally, the patients’ educational background also showed a difference in the caregivers’ overall life satisfaction. If patients had a college education or higher, the overall life satisfaction of caregivers reached 2.83 ± 0.72 (mean ± SD), followed by high-school graduates at 2.54 ± 0.78 (mean ± SD), and junior-high-school graduates at 1.60 ± 0.89 (mean ± SD) (*p* = 0.046) (Table 3).

### 3.4. Correlation of Physical Health, Household Income, Leisure and Social Activity, Family Relations, and Overall Life Satisfaction in General Status in Caregiver

As caregivers’ physical health increased, family economic level (r = 0.582, *p* = 0.001), leisure and social activities (r = 0.559, *p* < 0.001), family relationships (r = 0.488, *p* = 0.006), and overall life satisfaction (r = 0.634, *p* = 0.001) increased, indicating an increasingly positive correlation. On the contrary, depression showed a negative correlation (r = −0.555, *p* = 0.001). As the family economic level increased, leisure and social activities (r = 0.534, *p* = 0.002), family relationships (r = 0.528, *p* = 0.003), and overall life satisfaction (r = 0.583, *p* = 0.001) increased. Contrarily, depression showed a negative correlation (r = −0.429, *p* = 0.018). As leisure and social activities increased, there was a positive correlation between family relationships (r = 0.433, *p* = 0.017) and overall life satisfaction (r = 0.841, *p* = 0.001). Depression showed a negative correlation, decreasing to (r = −0.770, *p* < 0.001). As family relationships increased, overall life satisfaction increased (r = 0.597, *p* = 0.001) while depression showed a negative correlation (r = −0.466, *p* = 0.009). Finally, as overall life satisfaction increased, depression decreased (r= −0.646, *p* < 0.001), indicating an inverse correlation (Table 4).

### 3.5. Factor Association of Depression in Caregivers

The factors affecting caregivers’ depression were identified by using hierarchical multiple regression analysis, while controlling for related variables. In Model 1, the influence of caregiver depression was investigated by controlling for the caregivers’ physical health, family economic level, leisure and social activities, family relationships, and overall life satisfaction. It was found that the more leisure and social activities, the lower the depression of caregivers (B = −0.718, *p* = 0.001). In Model 2, the variable that showed a significant difference in caregiver depression in the univariate analysis was analyzed by adding the patients’ economic activity. It was found that the more leisure and social activities, the lower the depression of caregivers (B = −0.606, *p* = 0.004). Furthermore, the depression of caregivers increased (B = 0.438, *p* = 0.016) when patients did not engage in economic activity compared to economically active patients (Table 5).

### 3.6. Pathway Factors of Caregiver Depression

In exploring factors affecting caregiver depression, it was not possible to determine the multifaceted factors among the variables by using only hierarchical regression analysis. Therefore, it was analyzed in the form of a spider network through path model analysis.

**Figure 1:**
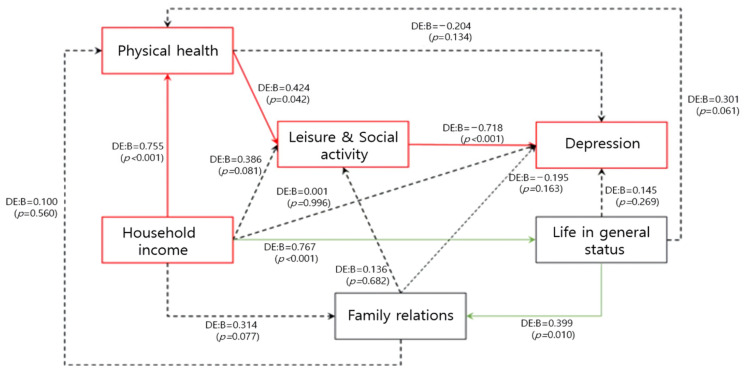
Pathway factors of caregiver’s depression. Red line: Mainly pathway factors of caregiver’s depression. Green line: Significant pathway, but no factors caregiver’s depression. Black dashed line: Not significantly pathway. DE: direct effect.

As a result, it was found that, as the family’s economic level increased, physical health increased (B = 0.755, *p* < *0*.001). Leisure and social activities increased (B = 0.424, *p* = 0.042) as physical health increased. Furthermore, as leisure and social activities increased, depression decreased (B = 0.718, *p* < 0.001). Regarding depression, physical health, family economic level, family relationships, and overall life satisfaction variables did not directly have a significant effect. When the family’s economic level increased (B = 0.767, *p* < 0.001), overall life satisfaction increased, and when overall life satisfaction increased, family relationships increased (B = 0.399, *p* = 0.010). However, family relationships did not significantly affect depression (Figure 1).

## 4. Discussion

Spinal cord injuries are increasing in frequency due to traffic accidents, slip and fall injuries, or recreational activities [17]. In our study, the age of patients with SCI at the time of the accident was mostly under 40, and such patients accounted for approximately 64% of all patients [18]. The increase in disability life expectancy due to a lower onset age compared to other chronic diseases affected not only the patient but also the family [19,20]. Due to the disability of a family member, families feel the burden of changing roles in anxiety and guilt, and they experience financial difficulties, an increased burden of care, and reduced social activities [21]. Meanwhile, the support and encouragement of a strong family play an essential role in the rehabilitation of the patient [22,23] and his/her smooth integration with society [20,22].

Care for patients with a spinal cord injury can affect the health of the family caregiver who is in charge of them, and studies on depression and accompanying health status of caregivers of spinal-cord-injury patients today can be quantified by using a variety of tools [21,22].

In this study, 63.3% of family caregivers under the age of 60 were women (80%), and the nature of the relationship with the SCI patient was as a spouse (63.3%), child (13.3%), or parent (10.0%). In many overseas studies [24,25,26], family caregivers were mostly women and spouses. These characteristics can be related to the characteristics of patients with SCI with a lower onset age compared to other chronic diseases and a higher frequency of accidents, such as falls or crushing trauma at work [17].

Seo et al. [7] found that families with spinal cord disorders have the greatest financial difficulties. Among the daily life-support services needed by the patients and their families to reduce the economic burden include support for assistive devices for patients, provision of welfare information and services, reductions in the costs of specialists to care for the patients, housing support, and mobility services, in this order of preference. As for social-activity support services, more than 71% of participants preferred support for additional family expenses, such as hospital expenses and rehabilitation treatment, as well as legal support (for expenses such as insurance payments). Moreover, support against physical fatigue and deterioration of the health of the family due to the daily support of patients was popular. In studies that measured the burden of caregivers supporting patients with SCI [24,27], the burden greatly increased according to the physical burden and time dependence of care. It was found that there is a difference in the subjectively felt physical burden between the primary caregiver and other family members, even within the same family member [27]. Our study also confirmed that the level of physical health felt by the primary caregiver was significantly lower than that of family members who did not care for the patient.

In a study of family caregivers of patients undergoing inpatient treatment for brain injury, there was a significant difference in the complaint rate of neck symptoms according to the length of care. In the shoulder, leg, and foot symptoms, there was a significant difference in symptom-complaint rates concerning gender, age, and length of care [28]. Bathing was the activity that caused the most complaints of musculoskeletal symptoms in nursing activities because it includes a series of procedures to move the patient, dressing and undressing, and protect the patient from falling on a slippery floor [29]. 

In this study, there were significant correlations between caregivers’ physical health, family economic level, leisure and social activities, family relationship, overall life satisfaction, and depression (*p* < 0.05). Factors showing a negative correlation with depression included physical health (r = −0.555, *p* = 0.001), family economic level (r = −0.429, *p* = 0.018), leisure and social activities (r = −0. 770, *p* < 0.001), family relationships (r = −0.466, *p* = 0.009), and overall living standards (r = −0.646, *p* < 0.001). Kuzu et al. [30] analyzed the direct and indirect effects of depression of the caregiver of a patient with SCI, the function of the patient with SCI, the stigma on the patient’s family due to the disability, and the burden of care. In that study, it was found that caregiver burden had a negative correlation with SCI patients’ residual function (r = −0.24, *p* < 0.01), a positive correlation with affiliate stigma (r = 0.36, *p* < 0.05), and a positive correlation with caregiver depression (r = 0.51, *p* < 0.01) [30]. In our study, the proportion of participants who answered that the burden of care was mainly economic was high, which is different from the study by Kuzu et al. [30].

The factor indicating a difference in the depression of family caregivers was patients’ economic activity (*p* = 0.013), and it was confirmed that caregiver depression increased when patients did not engage in economic activity (B = 0.438, *p* = 0.016). A study of caregivers of patients with chronic SCI revealed that there was a significant difference in mental health between the group in which SCI patients and their spouses were both unemployed and the group in which both were employed [31]. It was found that all employment conditions directly related to the patients increased their burden of care and deteriorated their quality of life, including mental health [31].

As a result of examining the effects of family caregivers on depression, it was confirmed that the more leisure and social activities the family caregivers engaged in, the lower their depression (B = −0.718, *p* = 0.001). Another study found that support through meetings with other family members and friends about the caregivers’ burden of care could be a protective factor against depression and physical health [27]. Among social supports, support from caregivers’ friends showed a negative correlation with care burden (r = −0.39, *p* < 0.001) and depression (r = −0.47, *p* < 0.001) [27]. 

Our study confirmed that the time devoted to caring by family caregivers (as the main caregivers) of patients with SCI increases and that limited leisure and social activities due to such care are influencing factors for depression. Family caregivers feel the burden of time due to providing help for SCI patients’ overall daily life and feel that they do not have time for themselves due to exhaustion. Consequently, they feel fatigue and depression in the caregiver role; in turn, their depressed feelings negatively affect the caregivers’ quality of life and make them perceive their situation more negatively. As these negative perceptions increase, caregivers may perceive their caregiving obligations to be more overwhelming, and a vicious cycle of increasing the burden of the caregiving role may be repeated. These results suggest that depression is an important interventional target for reducing the burden of care for family caregivers [27].

This study had several limitations. First, since caregivers of patients with SCI who were being treated as outpatients and inpatients at a local tertiary medical institution are subject to selection bias, there are limitations in generalizing the results for all family caregivers of patients with SCI. In addition, the risk of response bias cannot be excluded because the questionnaire used in this study depended on participants’ subjective self-report method. Third, while the measurement of the study results was cross-sectional and the relationship with the variables affecting the depression of family caregivers could be identified, the causal relationship could not be identified.

As psychological states such as depression of patients with SCI and their family caregivers can have a negative effect on their integrity, it is necessary to identify the factors affecting their psychological state. The needs for social services and policy support for the factors revealed through this study are urgent, and it should be recognized that support for family caregivers is a shortcut for the successful rehabilitation and smooth return to society of both caregivers and their patients. We hope that this study will be used as basic data to support policies for patients with spinal cord disorders and their caregivers.

## 5. Conclusions

This study comprehensively examined the factors affecting depression in caregivers and confirmed the demographic characteristics and subjective quality of life of caregivers providing support to patients with SCI. The main factors influencing family caregivers’ depression were leisure and social activities, as well as patients’ economic activity. It was confirmed that low levels of leisure and social activities were caused by the time constraints of the primary caregiver due to patient care. Thus, it would be helpful to establish a family support system to alleviate the depressive symptoms of family caregivers providing care for patients with SCI.

## Figures and Tables

**Table 1:** General characteristics (caregiver and patient).

Variables	Caregiver		Patient	
	*N*	%	*n*	%
Age				
≥60	11	36.7	12	40.0
<60	19	63.3	18	60.0
Sex				
Male	6	20.0	25	83.3
Female	24	80.0	5	16.7
Education level				
≥College	10	33.3	12	40.0
High school	10	33.3	13	43.3
≤Middle school	10	33.3	5	16.7
Economic activity				
Yes	13	43.3	11	36.7
No	17	56.7	19	63.3
Married status				
Married	25	83.3	23	23.3
Single	0	0.0	7	76.7
Other (separated, widowed)	5	16.7	0	0.0
Religion				
Yes	18	60.0	13	43.3
No	12	40.0	17	56.7
Relationship with the patient				
Spouse	19	63.3	-	-
Parents	3	10.0	-	-
Children	4	13.3	-	-
Other (grandparents, grandchildren, brothers, and sisters)	4	13.3	-	-
Patient care shifting				
Yes	7	23.3	-	-
No	23	76.7	-	-
Total	30	100.0	30	100.0

Note: Frequency test.

**Table 2:** Depression, physical health, household income, leisure and social activity, family relations, life-in-general status in caregiver.

Variables	Min–Max	M [SD]	Skewness +	Kurtosis ++
Depression	1–5	2.56 [0.82]	0.361	0.333
Physical health	1–4	2.70 [0.79]	−0.716	0.414
Household income	1–4	2.70 [0.75]	−1.010	0.977
Leisure and Social activity	1–4	2.50 [0.86]	−0.174	−0.491
Family relations	1–4	2.90 [0.76]	−0.842	1.269
Life-in-general status	1–4	2.50 [0.86]	−0.174	−0.491

Note: Descriptive statistics, M = mean, SD = standard deviation. Normality test: + skewness (not over than 2 absolute value), ++ kurtosis (not over than 4 absolute value).

**Table 3:** Comparison of caregiver’s depression, physical health, household income, leisure and social activity, family relations, and life-in-general status from the general characteristics (caregiver and patients).

Variables	Depression		Physical Health		Household Income		Leisure and Social Activity		Family Relations		Life-in-General Status	
	M [SD]	Z ^+^ or H ^++^ [*p*]	M [SD]	Z ^+^ or H ^++^ [*p*]	M [SD]	Z ^+^ or H ^++^ [*p*]	M [SD]	Z ^+^ or H ^++^ [*p*]	M [SD]	Z ^+^ or H ^++^ [*p*]	M [SD]	Z ^+^ or H ^++^ [*p*]
Caregiver												
Age												
≥60	2.58 [0.73]	−0.043 [0.966]	2.45 [0.69]	−1.540 [0.124]	2.73 [0.79]	−0.129 [0.898]	2.45 [0.93]	−0.138 [890]	2.91 [0.94]	−0.200 [0.841]	2.27 [0.91]	−1.193 [0.233]
<60	2.55 [0.89]		2.84 [0.83]		2.68 [0.75]		2.53 [0.84]		2.89 [0.66]		2.63 [0.83]	
Sex												
Male	3.33 [0.81]	−1.739 [0.082]	3.17 [0.81]	−2.209 [0.027]	3.00 [0.63]	−1.024 [0.306]	2.83 [0.75]	−0.995 [0.320]	3.17 [0.75]	−0.935 [0.350]	2.67 [0.82]	−0.359 [0.719]
Female	2.84 [0.72]		2.55 [0.72]		2.63 [0.77]		2.42 [0.88]		2.83 [0.76]		2.46 [0.88]	
Education level												
≥College	2.40 [0.78]	1.823 [0.402]	3.00 [0.67]	4.818 [0.090]	2.90 [0.57]	1.334 [0.513]	2.70 [0.82]	0.626 [0.731]	3.10 [0.57]	3.206 [0.201]	2.70 [0.82]	2.581 [0.275]
High school	2.36 [0.66]		2.90 [0.57]		2.80 [0.42]		2.50 [0.71]		3.10 [0.57]		2.70 [0.48]	
≤Middle school	2.93 [0.96]		2.20 [0.92]		2.40 [1.08]		2.30 [1.06]		2.50 [0.97]		2.10 [1.10]	
Economic activity												
Yes	2.26 [0.65]	−1.299 [0.194]	3.08 [0.49]	−2.282 [0.022]	3.00 [0.58]	−1.803 [0.071]	2.69 [0.75]	−0.826 [0.409]	3.15 [0.38]	−1.484 [0.138]	2.85 [0.69]	−1.852 [0.064]
No	2.79 [0.89]		2.41 [0.87]		2.47 [0.80]		2.35 [0.93]		2.71 [0.92]		2.24 [0.90]	
Married status												
Married	2.60 [0.89]	−0.752 [0.452]	2.64 [0.86]	−0.948 [0.343]	2.68 [0.80]	−0.166 [0.868]	2.44 [0.92]	−0.950 [0.342]	2.88 [0.03]	−0.162 [0.871]	2.44 [0.92]	−1.327 [0.342]
Single	-		-		-		-		-		-	
Other (separated, widowed)	2.35 [0.30]		3.00 [0.00]		2.80 [0.45]		2.80 [0.45]		3.00 [0.01]		2.80 [0.45]	
Religion												
Yes	2.59 [0.79]	−0.996 [0.319]	2.89 [0.76]	−1.587 [0.113]	2.83 [0.71]	−1.114 [0.265]	2.50 [0.86]	−0.113 [0.910]	3.00 [0.77]	−0.960 [0.337]	2.56 [0.86]	−0.406 [0.684]
No	2.52 [0.91]		2.42 [0.79]		2.50 [0.80]		2.50 [0.91]		2.75 [0.75]		2.42 [0.90]	
Relationship with the patient												
Spouse	2.64 [0.98]	1.080 [0.782]	2.53 [0.84]	6.983 [0.072]	2.58 [0.90]	1.573 [0.665]	2.53 [1.02]	2.049 [0.562]	2.53 [1.02]	1.167 [0.761]	2.47 [1.02]	3.371 [0.338]
Parents	2.62 [0.45]		3.67 [0.58]		3.00 [0.01]		2.00 [0.01]		2.00 [0.01]		2.00 [0.01]	
Children	2.51 [0.66]		2.50 [0.58]		3.00 [0.01]		2.50 [0.29]		2.50 [0.58]		2.50 [0.58]	
Other (grandparents, grandchildren, brother, and sister)	2.20 [0.18]		3.00 [0.01]		2.75 [0.50]		2.75 [0.50]		2.75 [0.50]		3.00 [0.01]	
Patient care shifting												
Yes	2.55 [0.71]	−0.025 [0.980]	2.57 [0.54]	−0.836 [0.403]	2.86 [0.38]	−0.499 [0.618]	2.29 [0.76]	−0.758 [0.448]	3.29 [0.49]	−1.568 [0.117]	2.43 [0.54]	−0.392 [0.695]
No	2.56 [0.87]		2.74 [0.86]		2.65 [0.83]		2.57 [0.90]		2.78 [0.80]		2.52 [0.95]	
Patients												
Age												
≥60	2.70 [0.66]	−0.700 [0.484]	2.75 [0.87]	−0.216 [0.829]	2.83 [0.72]	−0.912 [0.362]	2.42 [0.90]	−0.406 [0.684]	2.83 [0.84]	−0.369 [0.712]	2.42 [0.79]	−0.609 [0.542]
<60	2.47 [0.93]		2.67 [0.77]		2.61 [0.78]		2.56 [0.86]		2.94 [0.73]		2.56 [0.92]	
Sex												
Male	2.62 [0.84]	−0.752 [−0.452]	2.64 [0.81]	−0.853 [0.393]	2.64 [0.76]	−0.899 [0.369]	2.40 [0.87]	−1.424 [0.154]	2.84 [0.80]	−0.938 [0.318]	2.40 [0.87]	−1.424 [0.154]
Female	2.24 [0.77]		3.00 [0.71]		3.00 [0.71]		3.00 [0.71]		3.20 [0.45]		3.00 [0.71]	
Education level												
≥College	2.10 [0.67]	5.381 [0.068]	2.92 [0.52]	1.980 [0.371]	2.83 [0.58]	1.666 [0.430]	2.83 [0.72]	4.699 [0.095]	3.08 [0.67]	2.479 [0.289]	2.83 [0.72]	6.179 [0.046]
High school	2.78 [0.78]		2.69 [0.75]		2.77 [0.60]		2.46 [0.88]		2.92 [0.76]		2.54 [0.78]	
≤Middle school	3.10 [0.85]		2.20 [1.30]		2.20 [1.30]		1.80 [0.84]		2.40 [0.89]		1.60 [0.89]	
Economic activity												
Yes	2.04 [0.71]	−2.478 [0.013]	2.91 [0.54]	−0.953 [0.340]	2.91 [0.54]	−0.978 [0.328]	2.82 [0.75]	−1.400 [0.162]	3.09 [0.70]	−0.926 [0.355]	2.73 [0.79]	−0.872 [0.383]
No	2.86 [0.74]		2.58 [0.90]		2.58 [0.84]		2.32 [0.89]		2.79 [0.79]		2.37 [0.90]	
Married status												
Married	2.60 [0.89]	−0.752 [0.452]	2.70 [0.88]	−0.948 [0.343]	2.68 [0.80]	−0.166 [0.868]	2.44 [0.95]	−0.950 [0.342]	2.88 [0.83]	−0.162 [0.871]	2.44 [0.92]	−0.950 [0.342]
Single	-		-		-		-		-		-	
Other (separated, widowed)	2.35 [0.30]		3.00 [0.00]		2.80 [0.45]		2.80 [0.45]		3.00 [0.00]		2.80 [0.45]	
Religion												
Yes	2.59 [0.79]	−0.042 [0.967]	2.89 [0.76]	−1.141 [0.254]	2.83 [0.71]	−1.252 [0.211]	2.50 [0.86]	−1.339 [0.181]	3.00 [0.77]	−0.949 [0.343]	2.56 [0.86]	−1.540 0[.124]
No	2.52 [0.91]		2.42 [0.79]		2.50 [0.80]		2.50 [0.91]		2.75 [0.75]		2.42 [0.90]	
Total	2.56 [0.82]		2.70 [0.79]		2.70 [0.75]		2.50 [0.86]		2.90 [0.76]		2.50 [0.86]	

Note: M = mean, SD = standard deviation, The normality test was measured based on a nonparametric method.; ^+^ Mann–Whitney test (Z^+^): non-parametric test [2 group comparing], ^++^ Kruskal–Wallis test (H^++^)t: non-parametric test [≥3 group comparing], *p* = significant value.

**Table 4:** Correlation of physical health, household income, leisure and social activity, family relations, and life-in-general status in caregiver.

Variables	Physical Health	Household Income	Leisure and Social Activity	Family Relations	Life in General Status	Depression
	r [*p*]	r [*p*]	r [*p*]	r [*p*]	r [*p*]	r [*p*]
Physical health	1					
Household income	0.582 [0.001]	1				
Leisure and Social activity	0.559 [0.001]	0.534 [0.002]	1			
Family relations	0.488 [0.006]	0.528 [0.003]	0.433 [0.017]	1		
Life-in-general status	0.634 [<0.001]	0.583 [0.001]	0.841 [<0.001]	0.597 [0.001]	1	
Depression	−0.555 [0.001]	−0.429 [0.018]	−0.770 [<0.001]	−0.466 [0.009]	−646 [<0.001]	1

Note: Spearman Correlation analysis [non-parametric correlation], r = correlation coefficient, *p* = significant value.

**Table 5:** Factor association of depression in caregiver.

Variables	Model 1		Model 2	
	B [β]	t [*p*]	B [β]	t [*p*]
[Constants]	5.107 [-]	13.567 [<0.001]	3.995 [-]	6.318 [<0.001]
Physical health	−0.204 [−0.196]	−1.218 [0.235]	−0.196 [−0.189]	−1.285 [0.212]
Household income	0.001 [0.001]	0.006 [0.996]	0.018 [0.016]	0.111 [0.913]
Leisure and Social activity	−0.718 [0.200]	−3.584 [0.001]	−0.606 [−0.633]	−3.229 [0.004]
Family relations	−0.195 [0.161]	−1.209 [0.238]	−0.149 [−0.137]	−0.997 [0.329]
Life-in-general status	0.145 [0.231]	0.627 [0.537]	0.056 [0.059]	0.263 [0.795]
Patients Economic activity -No [ref: Yes]			0.438 [0.260]	2.607 [0.016]
R^2^, Adj R^2^	0.746, 0.693		0.806, 0.744	
F, *p*	14.068, <0.001		13.042, <0.001	

Note: Hierarchical regression analysis, R^2^ = model fit, Adj R^2^ = adjusted model fit, F = statistics value, *p* = significant value, B = unstandardized estimate, β = standard estimate.

## Data Availability

The datasets that were generated and analyzed during the current study are not publicly available but are available from the corresponding author upon reasonable request.
